# Tinnitus and event related potentials: a systematic review^☆^

**DOI:** 10.1016/j.bjorl.2019.09.005

**Published:** 2019-11-04

**Authors:** Andréia Aparecida de Azevedo, Ricardo Rodrigues Figueiredo, Norma de Oliveira Penido

**Affiliations:** a Universidade Federal de São Paulo (UNIFESP), São Paulo, SP, Brazil; b Otorrinolaringologia Sul Fluminense (OTOSUL), Volta Redonda, RJ, Brazil; c Faculdade de Medicina de Valença, Volta Redonda, RJ, Brazil

**Keywords:** Tinnitus, Event Related Potentials (ERP’s), P300, Long latency potentials, Auditory evoked potentials, Zumbido, Potenciais relacionados a eventos, P300, Potenciais de longa latência, Potenciais evocados auditivos

## Abstract

**Introduction:**

Tinnitus is sound perception in the absence of a sound source. Changes in parameters of latency and amplitude on the auditory event related potentials or long latency potentials waves have been cited in tinnitus patients when compared to a control group.

**Objective:**

To perform an assessment of scientific evidence that verifies the possibility of alterations in latency or amplitude of the waves of event related potentials in individuals with tinnitus.

**Methods:**

By using SciELO, Lilacs, ISI Web and PubMed, scientific databases, a review was performed. Articles published in English, Portuguese, French and Spanish that correlated tinnitus with changes in event related potentials were included in this review.

**Results:**

Twelve articles were located, however only eight fulfilled the criteria for inclusion.

**Conclusion:**

The sample of selected studies demonstrate that the long latency auditory evoked potentials related to events between the control and tinnitus patients showed some changes in latency and or amplitude in tinnitus patients. There are changes in event-related potentials when comparing patients with tinnitus and the control group. These changes take place considering the severity of tinnitus, tinnitus site of lesion, and capacity for changes after interventions. The event related potentials can help to determine the neurotransmitter involved in tinnitus generation and evaluate tinnitus treatments.

## Resumo

**Introdução:**

O zumbido é a percepção de um som na ausência de uma fonte sonora. Mudanças nos parâmetros de latência e amplitude nas ondas dos potenciais evocados auditivos relacionados a eventos ou potenciais de longa latência foram citadas em pacientes com zumbido quando comparados a um grupo controle.

**Objetivo:**

Realizar uma avaliação de evidências científicas que verifiquem a possibilidade de alterações na latência ou amplitude das ondas de potenciais evocados auditivos relacionados a eventos em indivíduos com zumbido.

**Método:**

Foi feita uma revisão a partir dos bancos de dados científicos SciELO, Lilacs, ISI Web e PubMed. Artigos publicados em inglês, português, francês e espanhol que correlacionavam zumbido com alterações nos potenciais evocados auditivos relacionados a eventos foram incluídos.

**Resultados:**

Foram localizados 12 artigos, porém apenas oito preencheram os critérios de inclusão.

**Conclusão:**

A amostra de estudos selecionados demonstra que os potenciais evocados auditivos de longa latência relacionados a eventos entre os pacientes-controle e com zumbido apresentaram algumas alterações na latência e/ou amplitude nos pacientes com zumbido. Há mudanças nos potenciais relacionados a eventos ao comparar pacientes com zumbido e o grupo controle. Essas alterações consideram a gravidade do zumbido, o local da lesão do zumbido e a capacidade de alterações após as intervenções. Os potenciais evocados auditivos relacionados a eventos podem ajudar a determinar o neurotransmissor envolvido na geração do zumbido e avaliar os tratamentos para o zumbido.

## Introduction

Tinnitus is defined as a sound perception in the absence of a sound source. It may cause distress and can lead to a chronic and debilitant condition. It is a frequent symptom; around 1/3 of the Western society population have experienced tinnitus at least once in their lives.^1^ Chronic and debilitating tinnitus affects 10% to 15% of the population. The severity of subjective tinnitus can only be assessed symptomatically, by patients, without an objective method to do so. In most cases the tinnitus perception increases in quiet environments.^2^ The subjective tinnitus and the perception of sound results from activity at the central nervous system without any vibratory activity within the cochlea mechanics or any external stimulus.^3^ Possible mechanisms for the origin of tinnitus in the cochlea or at the neuronal level have been discussed elsewhere.^4^, ^5^ However, the mechanisms that develop the level of stress caused by tinnitus remain unclear. Some authors believe that changes occur in the spontaneous neuronal activity which simulates the presence of external noise activity.^6^

Recent evidence suggests that neuronal and complex activity may be spontaneous or provoked by somatosensorial modulated stimuli, somatomotors and oculomotors.^7^ Most cases of tinnitus are associated with hearing loss, detected by the audiogram^8^ or by other more sensitive parameters.^9^ A key change following hearing loss is the tonotopic reorganization, in which neurons in the region of hearing loss in the primary auditory cortex (A1) begin to express a tuning from neighboring neurons, with their frequencies in the cortical map.^10^ Other possible factors arising from hearing loss include: changes in the imbalance between inhibition and excitation in the cortical auditory pathways, increased spontaneous activity of neurons in the central auditory system structures,^11^, ^12^ increased activity of these central structures^13^ and an increase in synchronous activity in cortical neurons affected by hearing loss.^14^ However, the contribution of these to the perception of tinnitus is not yet clear. An increased neural synchrony is due to restriction of the injured frequencies where human perception is also located.^15^, ^16^

In recent years, studies with long latency auditory potentials have been conducted exploring possible neural processes associated with tinnitus. The most important hypothesis^17^, ^18^, ^19^ concerning the pathophysiological mechanism of tinnitus involves an injury causing deafferentation of stimuli in thalamic cells. This would lead to a thalamic deactivation, deregulating thalamus cortical interactions and causing emerging tinnitus. Several authors have discussed the role of the thalamus in the pathophysiology of tinnitus and have pointed out the thalamus cortical dysrhythmia through Electroencephalography (ECG) and magneto encephalography as the main cause of tinnitus.

A reduction in the alpha and beta rhythms in the EEG would lead to an increase in activities of the areas involved, suggesting a hyperactivation of the left parietal lobe and inferior temporal gyrus and abnormal range activity of the medial temporal lobe in patients with tinnitus. A loss in the alpha and beta waves reinforce the hypothesis of increased cortical excitability in patients with tinnitus. Most studies show a trend to the cortical area, no matter the side of lesion.^20^

It is known that most of the auditory system is central. The auditory evoked potentials or related to events (ERPs) are used to examine the timing of discharge of the fibers of the auditory pathways and identify the presence of neuronal activities.^21^ The cognitive auditory potentials, including long latency, are often used to evaluate the function of higher cortical areas.^22^, ^23^ The N1 and N2 peaks are considered involuntary^24^ and the component P 300 a marker of voluntary attention. The identification of potential long latency in most literature studies is conducted using an unusual technique stimulus, Paradigm Oddball,^25^, ^26^, ^27^, ^28^, ^29^ which has02 different stimuli for generating the waves. Two types of stimulus are used: rare and frequent stimuli, which are perceived differently. The insula and upper cortical areas are the locations responsible for the generation of P300 auditory response. Since voluntary and involuntary attention are interconnected, integrated analysis of 03 long latency peaks N1, N2 and P3 are fundamental in the study of brain function.^30^

P300 is an event-related potential represented by a large and positive peak occurring 300 ms after the stimulus.^31^ For the P300, it is not clear whether the difference between the frequency to the rare and frequent stimuli is less than the discrimination threshold, though peripheral hearing loss can directly affect the P3 latency as well as the latency and amplitude of the waves N1 and N2.^32^

According to some authors, the P300 triggering is not influenced by moderate peripheral hearing loss and therefore should not prevent the use of this measure.^33^ The presence of N1 suggests the arrival of the stimulus to the auditory cortex. Studies on the extent of P300 changes related to gender and age have been described in the literature, although with no consensus. Latency increases with age and must be adjusted when analyzing the test result.^34^, ^35^, ^36^ The possibility of correlation between aspects of auditory behavior and observable physiological phenomena (cognitive potential ‒ P300) has increased the interest of professionals involved in the study of hearing disorders.^37^

## Objective

To assess the scientific evidences of changes in Latency and amplitude of the waves in Event related potentials in tinnitus patients.

## Methods

A systematic review of published articles about Tinnitus and Long Latency Potential was performed using the databases of PubMed, ISI Web (Web of Knowledge), SciELO and LILACS. For the search in PubMed and ISI Web, the strategy of advance search used the following keywords extracted from the Medical Subject Headings (MeSH): “Tinnitus”[Mesh] AND "Long Latency Potential “[Mesh] AND Event Related Potential” [MeSH Terms] AND (English [lang] OR Spanish [lang] OR French [Lang] OR Portuguese [lang]). For the LILACS and SciELO databases, we utilized the keywords indexed in the Health and Sciences Keywords using the following strategy: Tinnitus AND Long latency potential Or Event Related Potentials” The basic research form was used with the term AND in order to relate the words (Long latency potential and tinnitus) and the trucking sign was used in order to search for words with the same root word of the keyword “Long Latency potential” OR “Event related potential”. The choice of using the keyword “Long latency Potential” AND “Event Related Potential was justified to create a more comprehensive review, considering the fact that some articles only employ the term “Long latency potential” or” Event Related Potential “as a keyword. Included in the search was a series of patient studies in which the patients were 18 years or older, published in English, French or Portuguese, which associated tinnitus and long latency potential. Letters to the Editor and Case Reports were not included in the search. Two reviewers, organized under the following subject titles, carried out data extraction from the selected papers: Authors, Year of Publication, Sample Size, Type of Study, Instruments Used, and Principle Results of Associations between tinnitus and long latency potential.

The criterion for inclusion of articles in this systematic review was designed to compare the waves between the tinnitus group and control, without any intervention. The studies were performed with different methods and changes in relative amplitude and latency in the waves, showing differences when comparing control group and tinnitus patients’ group.

## Results

The systematic review produced a total of 12 articles from which 11 were found in PubMed and 01 in LILACS. The articles had publication dates ranging from 1991 to 2015. Access to the articles was conducted on line through Coordination for Improvement of Personnel with Higher Education (CAPES) website by the researchers using BIREME library (Bibliotheca Regional de Medicine) so as to obtain copies of the published journals. Additionally, some published articles were made available through the personal library of the authors of this report.

Among the 12 articles there were 9 cross sectional studies, 2 prospective studies and 1 retrospective study. Among the population studies 8 studies compared the amplitude and latency of N1, P2, N2 and P3 between tinnitus and after Repetitive Transcranial Magnetic Stimulation (rTMS), 1 compared changes in ERPs before and after sensory training therapies between tinnitus patients and control and 1 compared tinnitus patients’ distress (high and low) and the degree of attention with ERP. Table 1 shows the main interest parameters of the selected articles. Of the 12 studies four articles were excluded because there was an intervention and or different criteria.^38^, ^39^, ^40^, ^41^

**Table 1:** Main interest parameters of the selected articles.

Authors and year	Study design	Sample size (n)	Type of sample	Primary outcome (tinnitus x long latency potential
Shiraishi et al., 1991	Transversal	40	Compare the contingent negative variations recording and examining the negative and positive potentials (N100 and P200) in Tinnitus Patients and control.	Contingent negative variation amplitude was significant in patients than in controls (*p* < 0.05, Anova) but no significant differences were observed in N100 and P300 latency and Amplitudes for the two groups. However amplitudes recorded in Tinnitus group tended to be smaller.
Attias et al., 1993	Transversal	24	Explore a possible deficit in auditory central neural activity with Noise induced hearing loss. ERPs^a^ and Reaction Time (RT) were recorded from tinnitus patients and controls.	ERP amplitudes (Wave N100, P200 And P300) in tinnitus Patients were significantly lower than in controls in all resting paradigms. No differences were found in ERPs Peak latencies.
Jacobson et al, 2003	Case control transversal study	63	Long latency in Tinnitus and Normal subjects.	Tinnitus patients presented smaller amplitudes for N100.
Santos et al., 2010	Prospective study, case transversal control	60	Investigate the Event Related Potential N100, P200 and P300 in Patients exposed to occupational noise with and without Tinnitus and verify if there is an association with the Tinnitus localization side.	It was noticed occurrence of LLAEP^b^ changes in tinnitus patients and the most commonly type was the increase in latency values and it was Noticed also an association between the side of LLAEP N100, N200 and P300 component changes in bilateral Tinnitus.
Said, 2011	Transversal	90	Study whether differences exist in ABR and/or ERPs parameter in 2 groups: sensorineural hearing loss with and without Tinnitus and control.	Higher Prevalence of ABR^c^ abnormalities in Tinnitus patients in comparison to the control or SNHL^d^ without Tinnitus. The results of ERPs it was found an increase in Latency (N100, P200 and P300) and an amplitude reduction (P200, P300).
Yang et al., 2013	Prospective study	36	The ERPs were compared in Tinnitus and normal subjects and the alterations in ERPs before after repetitive Transcranial Magnetic Stimulation (rTMS).	Before the rTMS, control group presented a larger N100 (deviant) and Tinnitus Group a smaller N100 (standard). After rTMS, Tinnitus Group presented a larger N100 (deviant).
Elmorsy et al., 2013	Case transversal control	62	Investigate and measure auditory P300 response in patient with idiopathic tinnitus and to compare it with normal subjects.	Auditory P300 event related potentials to tone burst stimuli showed peak amplitudes overall reduced for Idiopathic Tinnitus Subjects (IST) than to Normal subjects. P300 peak latencies were not statistically significant.
Houdayer et al., 2015	Retrospective study 2009‒2013	34	Asses the level of involvement of different brain circuits in normoacoustic Tinnitus suffers compared non Tinnitus suffers using Electroencephalography (EEG) and ERPs.	No significant differences between suffers and control in P300 Peak and latency. Shorter Latency of N100 in tinnitus sufferers (rare and frequent) and shorter latency of the P200 (rare).

a ERPs, evocated related potentials.

b LLAEP, long latency auditory evoked potential.

c ABR, auditory brainstem response.

d SNHL, sensorineural hearing loss.

Eight papers located met the review´s criteria and were included in the study. The studies selected demonstrated a possible association between tinnitus and long latency potentials. As the differences varied from one study to another and statistically there was not all the necessary data described in the studies selected, it was impossible to realize a Meta-Analysis.

Shiraishi et al. compared the contingent negative variations recording and examined the negative and positive potentials (N1 and P3) in tinnitus patients and control. Contingent Negative Variation (CNV), also referred as an expectance wave, is a slow negative cortical potential shift appearing in the front central regions of the human scalp. Contingent negative variation amplitude was more significant in patients than in controls but no significant differences were observed in N100 and P300 latency and amplitudes for the two groups. However, amplitudes recorded in tinnitus group tended to be smaller.^42^

Santos reported a reduction of N1 latency in patients with bilateral tinnitus; the amplitude N1-P2 was increased in the ear with unilateral tinnitus.^43^

Haldy et al. compared the event-related potentials in patients with tinnitus and control group, checking for previous and subsequent changes of transcranial stimulation and compared with innitus handicap inventory. They found previously rTMS a reduced amplitude for N1, MMN and LDN components in tinnitus patients compared to the control group. After repetitive Transcranial Magnetic Stimulation (rTMS) tinnitus patients showed an increasein amplitude of P1, MMN and LDN. After rTMS treatment, tinnitus patients showed increased N1 response to deviant stimuli and larger MMN and LDN compared with pre-treatment. This situation can confirm the different sites involved in tinnitus patients and may in the future justify the subtypes of tinnitus.^44^

Eman et al. studied the electrophysiological differences in sensorineural hearing loss patients with and without problem-tinnitus and changes were found. In tinnitus subgroups for both women and men there were altered results in P2, P3 latency and P3 amplitude components when compared with the control group of the same sex. Comparing the group of women and the control group it is revealed that the group of women with tinnitus presents higher latencies values N1, P2, N2 and P3. Between the woman group with and without innitus there were statistically significant differences in the latencies of N1, P2, and P300. The same happened with the male group. When compared with the tinnitus male group with the control group there was in all auditory ERP a higher latency value: N1, P2, N2, P3. Also, when this was compared with the male subgroup without tinnitus we found significant statistically higher mean latency values for (N1, P2, and P3). As for the amplitude values there was no difference between the groups of hearing loss without tinnitus and the control group, however a reduction in the amplitude of P2 and P3 was demonstrated when compared to the hearing loss with tinnitus and the control group. Furthermore, it was demonstrated that comparing hearing loss with and without tinnitus there is lower P3 amplitude for the Tinnitus group.^45^

Attias et al. explored a possible deficit in auditory central activity in tinnitus with Noise Induced Hearing Loss (NIHL) and also subjects with a history of repeated noise exposure and NIHL without tinnitus. Auditory Event Related Potentials (ERP) and Reaction Time (RT) were recorded. The audiograms in both groups demonstrated typical NIHL. ERP amplitudes, wave N1, P2 and P3 in tinnitus patients were significantly lower than in controls in all resting paradigms. No differences were found in ERP Peak latencies. No differences between the controls and tinnitus patients reflecting auditory nerve and brain stem functioning; both populations displayed similar values to measures. In contrast the ERP results of tinnitus differed significantly from the controls. The ERP components N1, P2 e P3 in response to a variety of stimuli were associated with lower amplitudes. No differences were observed in component latencies. Attias et al concluded that the speed of these cognitive processes in tinnitus patients is not affected.^46^

Jacobson et al. studied long latency in tinnitus and normal subjects. In this investigation passive and selective auditory attention were employed. They found that the N1 amplitude in the attend condition was significantly larger than N1 amplitude in the ignore condition subjects.

Across listening conditions, attend and ignore, N1 amplitude was smaller for patients with tinnitus. This finding has been interpreted in the past as evidence for the presence of adaptive brain process that occurs for tinnitus patients. That is, the presence of continuous afferent signals (i.e. tinnitus) generated subcortically results in upstream adaptation in the manner by which all auditory signals are processed. This would have the effect of decreasing N1 amplitude for tinnitus subjects.^47^

Shawky et al. investigated and measured auditory P300 response in patients with idiopathic tinnitus and compared it with normal subjects. Auditory P300 event related potentials to tone burst stimuli showed P300 peak amplitudes overall reduced for idiopathic tinnitus subjects (IST) than in normal subjects. P300 peak latencies were not statistically significant.^48^

Houdayer et al. accessed the level of involvement of different brain circuits in normacoustic tinnitus sufferers compared non-tinnitus sufferers, using EEG and ERPs. The findings showed shorter latencies of N100 in tinnitus sufferers, both for the rare and frequent stimulus condition and a shorter latency of P2 only in rare conditions. Regarding P300, the values did not differ significantly between groups.^49^

Eight articles with similar study criteria were selected as shown in Table 1.

## Discussion

There might be a correlation between tinnitus and biological changes in long latency potential. Auditory evoked potentials are used to examine the timing of the discharge of auditory fibers and identify the presence of neuronal activity. According to some reports there is a reduction of alpha rhythms, beta and gamma, which reinforces the hypothesis of cortical dysfunction thalamus-linked in tinnitus patients. Most of these authors have discussed the thalamic arrhythmias as a possible cause of tinnitus. The majority of the studies agree that there is an association between tinnitus and an alteration on latency and or amplitude of N1, but some studies also found reduced amplitude of P300 in patients with tinnitus showing that perhaps there is an inhibition in the attention of the outside stimulus in tinnitus patients. There is objective evidence that attention in patients with high tinnitus- related distress is focused more to the tinnitus as compared to patients with low distress. It therefore might be useful to apply this knowledge in neurofeedback-based therapies of tinnitus, aiming at maximizing the ability to shift attention away from the tinnitus. Attention training in order to shift attention away from the tinnitus is already part of frequently used cognitive- behavioral therapies of tinnitus.

It has been observed that in most tinnitus patients there is a clear difference related to latency and or amplitude of the event related potentials when compared to the control group.

After repetitive Transcranial Magnetic Stimulation (rTMS) tinnitus patients showed increased amplitude of P1, MMN and LDN. After rTMS treatment, tinnitus patients showed increased N1 response to deviant stimuli and larger MMN and LDN compared with pre-treatment. This situation can confirm the efficacy of a treatment.

The tinnitus can be a third stimulus and can influence every wave of the long latency potentials, even the N1 and N2 peaks that are considered involuntary and the component P300 a marker of voluntary attention. The presence of continuous afferent signals (i.e. tinnitus) generated subcortically results in an upstream adaptation in the manner by which all auditory signals are processed. This would have the effect of long latency potentials for tinnitus subjects. There is a possible adaptation brain process that occurs for tinnitus patients. The auditory long latency potentials register the discharge of the fibers of the auditory pathways and the presence of neuronal activities. It is possible that some patients when performing the long latency auditory potential can perceive 01 frequent stimulus, one continuous stimulus and one rare stimulus in odd ball paradigm and this fact can create difficulties for the patient to pay attention to the rare stimulus and can influence the latency and the amplitude of the long latency waves. The patient can be focused on tinnitus due to the inhibitory neurotransmitters that can be acting and inhibiting the outsider stimulus, interfering with the patient’s personality and attention and making patient stress a severity factor to their lives. It is known that tinnitus patients avoid a silent environment due to the worsening of tinnitus perception, and this happens because patients are able to pay more close attention to the tinnitus when there is no other noise. This situation suggests that there is an attention to the interior sound (i.e. tinnitus). This suggests that the attention to the tinnitus may be a dominant factor of tinnitus complaints.

## Conclusion

The sample of selected studies demonstrate that the long latency auditory evoked potentials related to events between the control and tinnitus patients showed some changes in latency and or amplitude in tinnitus patients. There are changes in event-related potentials when comparing patients with tinnitus and the control group. These changes take place considering the severity of tinnitus, tinnitus site of lesion, and capacity for changes after interventions. The event related potentials can help to determine the neurotransmitter involved in tinnitus generation and suggest tinnitus treatments.

## Conflicts of interest

The authors declare no conflicts of interest.
